# Beyond Element-Wise Interactions: Identifying Complex Interactions in Biological Processes

**DOI:** 10.1371/journal.pone.0006899

**Published:** 2009-09-23

**Authors:** Christophe Ladroue, Shuixia Guo, Keith Kendrick, Jianfeng Feng

**Affiliations:** 1 Department of Computer Science and Mathematics, Warwick University, Coventry, United Kingdom; 2 Mathematics and Computer Science College, Hunan Normal University, Changsha, People's Republic of China; 3 Laboratory of Behaviour and Cognitive Neuroscience, The Babraham Institute, Cambridge, United Kingdom; 4 Department of Computer Science and Mathematics, Warwick University, Coventry, United Kingdom and Centre for Computational System Biology, Fudan University, Shanghai, People's Republic of China; Mount Sinai School of Medicine, United States of America

## Abstract

**Background:**

Biological processes typically involve the interactions of a number of elements (genes, cells) acting on each others. Such processes are often modelled as networks whose nodes are the elements in question and edges pairwise relations between them (transcription, inhibition). But more often than not, elements actually work cooperatively or competitively to achieve a task. Or an element can act on the interaction between two others, as in the case of an enzyme controlling a reaction rate. We call “complex” these types of interaction and propose ways to identify them from time-series observations.

**Methodology:**

We use Granger Causality, a measure of the interaction between two signals, to characterize the influence of an enzyme on a reaction rate. We extend its traditional formulation to the case of multi-dimensional signals in order to capture group interactions, and not only element interactions. Our method is extensively tested on simulated data and applied to three biological datasets: microarray data of the *Saccharomyces cerevisiae* yeast, local field potential recordings of two brain areas and a metabolic reaction.

**Conclusions:**

Our results demonstrate that *complex* Granger causality can reveal new types of relation between signals and is particularly suited to biological data. Our approach raises some fundamental issues of the systems biology approach since finding all complex causalities (interactions) is an NP hard problem.

## Introduction

Uncovering the existence and direction of interactions between elements of a set of signals remains a difficult and arduous task that one has to face if one wants to understand the mechanisms at work in most biological phenomena and make full use of the high-throughput experimental data that is now more and more available. A network structure carefully inferred from experimental data could provide us with critical information about the underlying system of investigation and is an important topic in systems biology. For example, high-throughput data from gene, metabolic, signaling or transcriptional regulatory networks, contain information about thousands of genes or proteins. *Group* interactions are common in these networks, as nodes may work cooperatively or competitively to accomplish a task. Another type of interaction is one where an element has some control on the interaction between two others. We call “*complex*” these types of interactions, to distinguish them from the more usual pairwise, element-to-element relations traditionally assumed.

**Figure 1:**
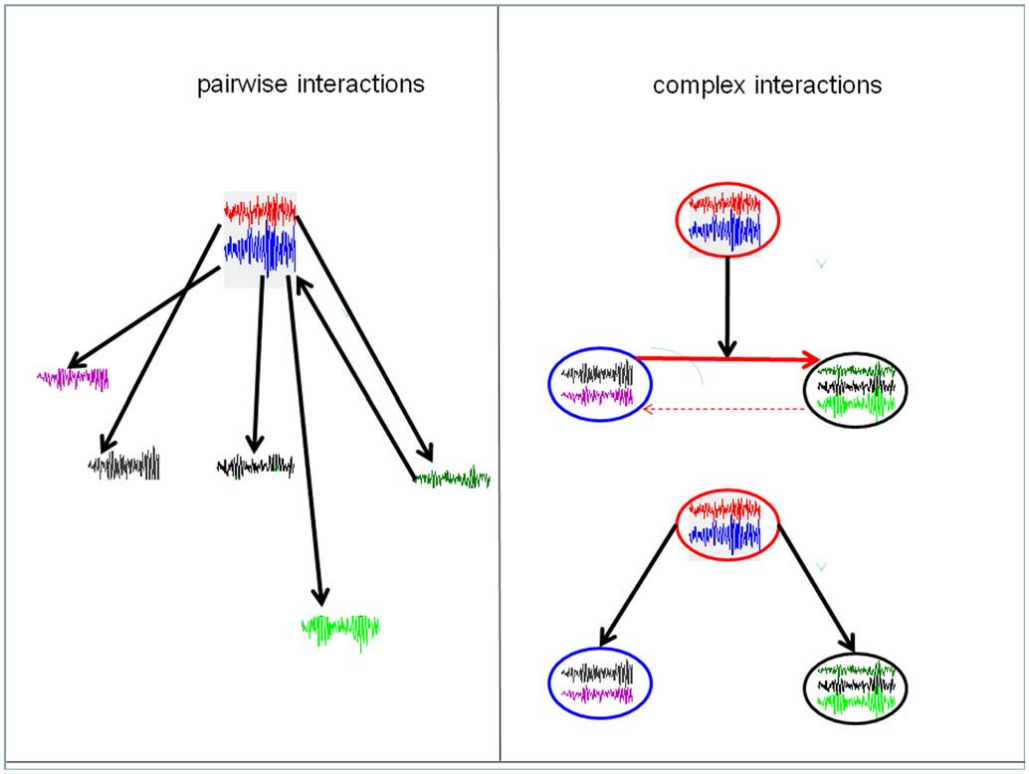
A schematic plot of the complex interactions. Each time trace (node) is the activity of a gene, protein, substance etc. A circle is a complex comprising of nodes. Left panel is the interactions among nodes. Right panel, the top complex can exert its influence on the rate between two complexes (top), or on the complexes themselves (bottom).

The complex interactions differ considerably from the interactions among single nodes that have been extensively studied in the past decades.For example, one can picture a situation where two nodes do not interact with a third one when considered invidually but do once considered together (cooperation). A more subtle example is the case of a chemical reaction from a substrate S to a product P catalysed by some enzyme E. The enzyme acts on the reaction rate from S to P but not from P to S. Being able to identify such interations from observed data is obviously an interesting and challenging task (see Fig. 1). To fully understand the properties of a network, whether it is a gene, a protein or a neuronal network, it is therefore of prominent importance to consider complex interactions.

This issue has been realized, and it has been tested intensively in many experiments. For example, LOF (loss of function) experiments are performed for double, triple and quadruple mutations. Two commonly used computational approaches to explore the experimental data and recover the interactions between units in the literature are Bayesian networks [1] and Granger causality [2]–[6]. However, to the best of our knowledge, no systematic approach has been developed to take this issue into account. Here we adopt the Granger causality approach. The concept of the Granger causality – originally introduced by Wiener [7] and formulated by Granger [2] – has played a considerably important role in investigating the relationship among stationary time series. Specifically, given two time series, if the variance of the prediction error for the second time series at the present time is reduced by including past measurements from the first time series in the (non)linear regression model, then the first time series can be said to (Granger-)cause the second time series. In other words, 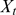 is a (Granger-) cause of 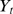 if 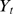 is better predicted when 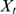 is taken into account. Granger causality thus provides two types of information at once: the magnitude of the interaction – a non-negative number, with 0 meaning an absence of interaction, and its direction – the measure is not symmetric in its arguments. Geweke's decomposition of a vector autoregressive process [8]–[11] led to a set of causality measures which have a spectral representation and make the interpretation more informative and useful: the spectrum of Granger causality shows at which frequencies the interaction takes place.

To tackle the issue of complex interactions we extend the pairwise Granger causality and the partial Granger causality we proposed in [12] to *complex Granger causality*, both in the time and frequency domains. The previous methods were limited to the study of interactions between one-dimensional signals. Our extension accepts multi-dimensional data and thus defines Granger Causality between groups of signals. We apply our approach to simulated and experimental data to validate the efficiency of our approach. With simulated data, we first demonstrate that our *complex Granger causality* can reliably detect group interactions, both in the time and frequency domains. We then show how Granger causality can detect the overall larger effect of two signals of little influence. Spurious interactions can be mistaken for genuine ones when the interaction between two groups is completely mediated by a third one. We extend the *complex Granger causality* to *partial complex Granger causality* which removes the influence of the mediating group and thus provides a more accurate measure of the connection between the two groups.

Complex Granger causality is then applied to three different biological problems in order to illustrate its ability to capture these new types of interactions (group-to-signal, group-to-group and group-to-interaction). First, we use yeast cell-cycle microarray data to compare results obtained when complexes-to-single gene connections are not taken into account and when they are. Next, we use complex Granger causality to study the connections between brain areas and compare the results obtained from considering individual signals alone or region averages. Finally, we consider a well-known metabolic reaction and show that our method can capture the effect of an enzyme over a chemical reaction rate.

## Methods

### Complex Granger causality

Granger causality quantifies the strength of the connection from a signal 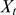 to a signal 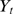. Formalised by Granger ([2], [3]), it consists in comparing the magnitude of the errors before and after including 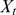 for predicting 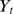. This quantity, often noted 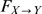, is a non-negative number with the minimum 0 denoting absence of connection. Granger Causality is traditionally only defined for one-dimensional time-series. Here, we extend its usual formulation to the case of multi-dimensional signals. A frequency domain formulation is also possible and produces a spectrum, rather than a single value, giving the frequencies at which the interactions occur.

#### Time Domain Formulation

Consider two multiple stationary time series 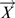 and 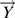 with 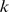 and 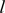 dimensions respectively. Individually and under fairly general conditions, each time series has the following vector autoregressive representation
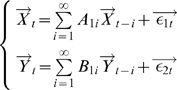
(1)where 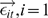 are normally distributed random vectors with 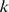 and 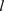 dimensions. Their contemporaneous covariance matrix are 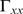 and 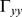 with trace being denoted by 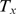 and 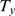 respectively. The value of 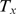 is non-negative and equals to the summation of all eigenvalues of 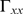, which measures the accuracy of the autoregressive prediction of 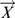 based on its previous values, whereas the value of 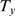 represents the accuracy of predicting the present value of 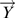 based on previous values of 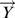.

Jointly, they are represented as
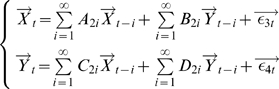
(2)where the noise terms are uncorrelated over time and their contemporaneous covariance matrix is
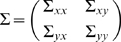
(3)


The submatrices are defined as 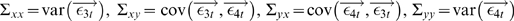 If 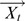 and 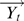 are independent, the coefficient matrices 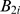 and 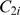 are zero, 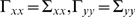, 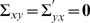. The traces of 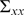 and 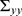 are denoted by 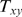 and 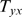 respectively. Consider eq. (2), the value of 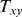 represents the accuracy of predicting the present value of 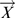 based on previous values of both 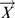 and 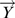. According to the causality definition of Granger, if the prediction of one time series is improved by incorporating past information of the second time series, then the second time series causes the first process. We extend them to multiple dimensional cases. If the trace of the prediction error for the first multiple time series is reduced by the inclusion of the past of the second multiple time series, then a causal relation from the second multiple time series to the first multiple time series exists. We quantify this causal influence by
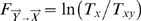
(4)


It is clear that 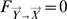 when there is no causal influence from 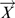 to 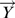 otherwise 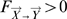. Moreover, if 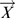 and 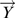 are one-dimensional, the definition reduces to the traditional Granger Causality and thus is consistent with the latter.

Note that in constrast with our previous extension of Granger causality ([12]), the complex Granger causality is now formulated in terms of the trace – and not the determinant – of matrices, for numerical stability and more theoretical considerations (see discussion below).

#### Frequency Domain Formulation

Time series contain oscillatory aspects in specific frequency bands. It is thus desirable to have a spectral representation of causal influence. We then consider the frequency domain formulation of complex Granger causality. Rewriting eqs. (2) in terms of the lag operator, we have:
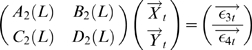
(5)where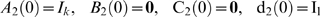. Fourier transforming both sides of eqs.(5) leads to
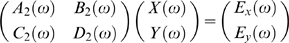
(6)where the components of the coefficient matrix are
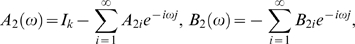


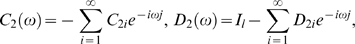



Recasting eq.(6) into the transfer function format we obtain
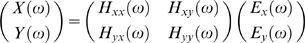
(7)the components of 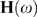 are
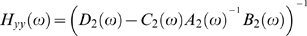


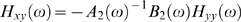


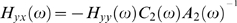


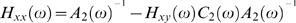



After proper ensemble averaging we have the spectral matrix
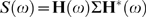
where 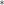 denotes the complex conjugate and matrix transpose, and 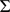 is defined in eq. (3).

To obtain the frequency decomposition of the time domain causality defined in the previous section, we look at the auto-spectrum of 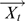


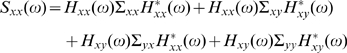
(8)


Note that the elements of the diagonal of 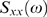 are reals. The trace of both sides can be represented as
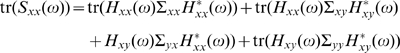
(9)


We first consider a simple case 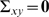. The second term of the right side of eq.(9) is zero. We have
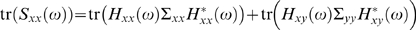
(10)which implies that the spectrum of 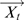 has two terms. The first term, viewed as the intrinsic part, involves only the noise term that drives 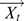. The second term, viewed as the causal part, involves only the noise term that drives 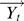.

When 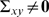 we can normalize eq. (6) by multiplying the following matrix
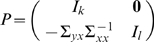
(11)to both sides of eq.(6). The result is
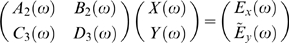
(12)where 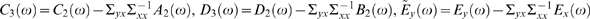 From the construction it is easy to see that 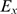 and 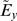 are uncorrelated. The variance of the noise term for the normalized 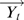 equation is 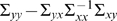. The new transfer function 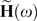 for eq.(12) is the inverse of the new coefficient matrix
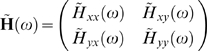
(13)where
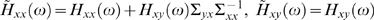


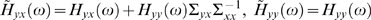



Note that 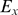 and 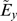 are uncorrelated. Following the same steps of eq.(10), the spectrum of 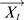 is found to be
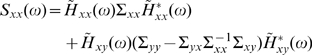
(14)


The trace of both sides can be represented as
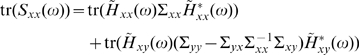
(15)


Here the first term is interpreted as the intrinsic power and the second term as the causal power of 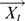 due to 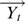. We define the causal influence from 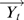 to 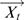 at frequency 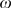 as
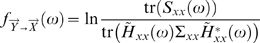
(16)


### Partial Complex Granger causality

In this section, we define *partial* Complex causality to remove the influence of a mediating group from the connection between two others. This approach allows us to discard indirect interactions between groups and get a more accurate measure of the relation between groups. As in the case of complex Granger Causality, it is defined both in the time and frequency domains.

#### Time Domain Formulation

Consider three multiple stationary time series 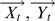 and 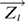 with 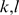 and 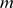 dimensions respectively. We first consider the relationship from 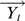 to 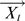 conditioned on 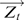. The joint autoregressive representation for 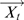 and 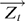 can be written as
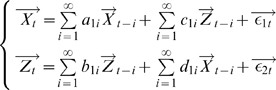
(17)


The noise covariance matrix for the system can be represented as
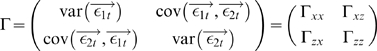
where 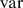 and 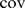 represent variance and covariance respectively. Extending this representation, the vector autoregressive representation for a system involving three time series 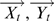 and 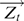 can be written in the following way.
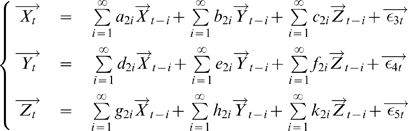
(18)


The noise covariance matrix for the above system can be represented as
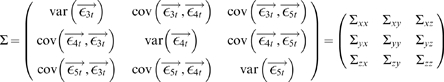
where 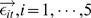 are the prediction errors, which are uncorrelated over time. The conditional variance 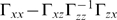 measures the accuracy of the autoregressive prediction of 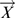 based on its previous values conditioned on 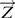 whereas the conditional variance 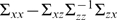 measures the accuracy of the autoregressive prediction of 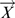 based on its previous values of both 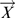 and 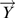 conditioned on 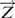. The traces of the matrix 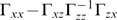 and the matrix 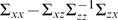 are denoted by 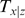 and 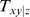 respectively. We define the partial Granger causality from vector 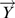 to vector 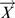 conditioned on vector 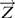 to be
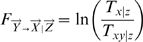
(19)


#### Frequency Domain Formulation

To derive the spectral decomposition of the time domain partial Granger causality, we first multiply the matrix
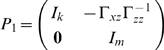
(20)to both sides of eq. (17). The normalized equations are represented as:
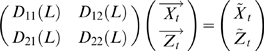
(21)with 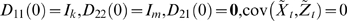 we note that 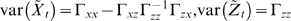. For eq. (18), we also multiply the matrix
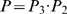
(22)where
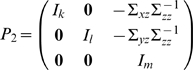
(23)and
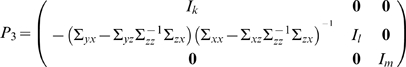
(24)to both sides of eq.(18). The normalized expression of eq. (18) becomes
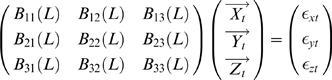
(25)where 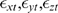 are independent and their variances 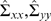 and 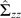 with
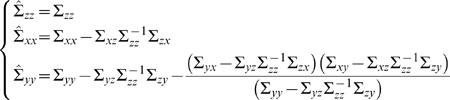



After Fourier transforming eq. (21) and eq. (25), we can rewrite these two equations in the following expression:
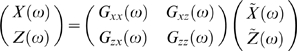
(26)and
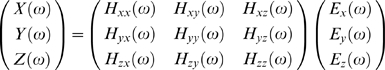
(27)


Note that since 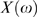 and 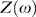 from eq. (26) are identical with that from eq. (27), we thus have
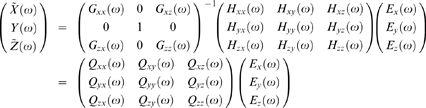
(28)where 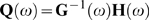. Now the power spectrum of 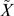 is
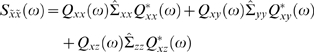
(29)


The trace of both sides of eq. (29) can be represented as
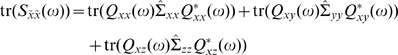
(30)


Note that 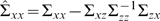
The first term can be thought of as the intrinsic power eliminating exogenous inputs and latent variables and the remaining two terms as the combined causal influence from 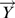 mediated by 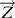. This interpretation leads immediately to the definition
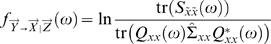
(31)


In previous studies, we showed that by the Kolmogorov formula ([11]) for spectral decompositions and under some mild conditions, the Granger causality in the frequency domain and in the time domain satisfy
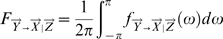
(32)


All our numerical simulations and applications on real data strongly suggest this is still the case with the present extension of the definition. However, whether it is true in general remains a conjecture at this stage.

## Results

### Simulated data: pairwise complex interaction

#### Example 1

Suppose that 2 simultaneously generated multiple time series are defined by the equations
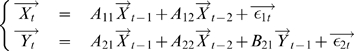
where 
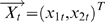
 is a 2-dimensional vector, 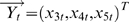 is a 3-dimensional vector, 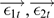 are normally distributed random vectors. The coefficient matrices are
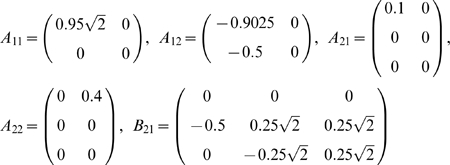



**Figure 2:**
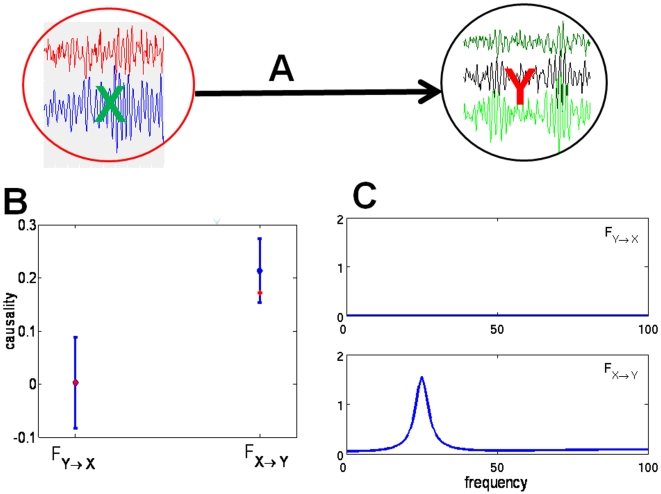
Application of Complex Granger Causality to simulated data. (A). Time series considered in Example 1. The underlying causal relationship is represented by an arrow. (B) Comparison between the time domain pairwise Granger causality and the frequency domain pairwise Granger causality. The partial complex Granger causality and its 95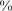 confidence interval after 1000 replications are shown in blue. The summation over a frequency range of the corresponding frequency-domain formulation is shown in red. (C) the corresponding spectra in the frequency domain.

We perform a simulation of this system to generate a dataset of 2000 data points with a sample rate of 200 Hz. The time courses of the two vectors 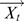 and 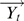 are plotted in Fig. 2 (**A**) (inside ovals). From the model, 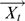 is clearly a direct source of 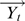, which in turn does not have any influence on 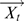, as represented in Fig. 2.

Fig. 2(**B**) presents a comparison between the time domain complex Granger causality and the frequency domain complex Granger causality (see Fig. 2 (**C**) for details). Blue error bars are the value of the complex Granger causality calculated in the time domain. The standard errors are estimated with a bootstrap of 1000 replications. Red error bars are the summation (integration) of the complex Granger causality for frequencies in the range of 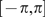. Fig. 2 (**C**) shows the results obtained in the frequency domain. As expected, Fig. 2 (**C**) demonstrates that the decomposition in the frequency domain fits very well with the Granger causality in the time domain. The direct causal link from 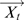 to 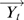 is clearly seen, as well as the absence of interaction from 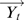 to 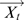.

This example demonstrates that complex Granger causality can detect interactions between groups. In the next example, we show how a group of signals can have a significant impact on another signal, even if individual interactions are too weak to be detected.

#### Example 2

**Figure 3:**
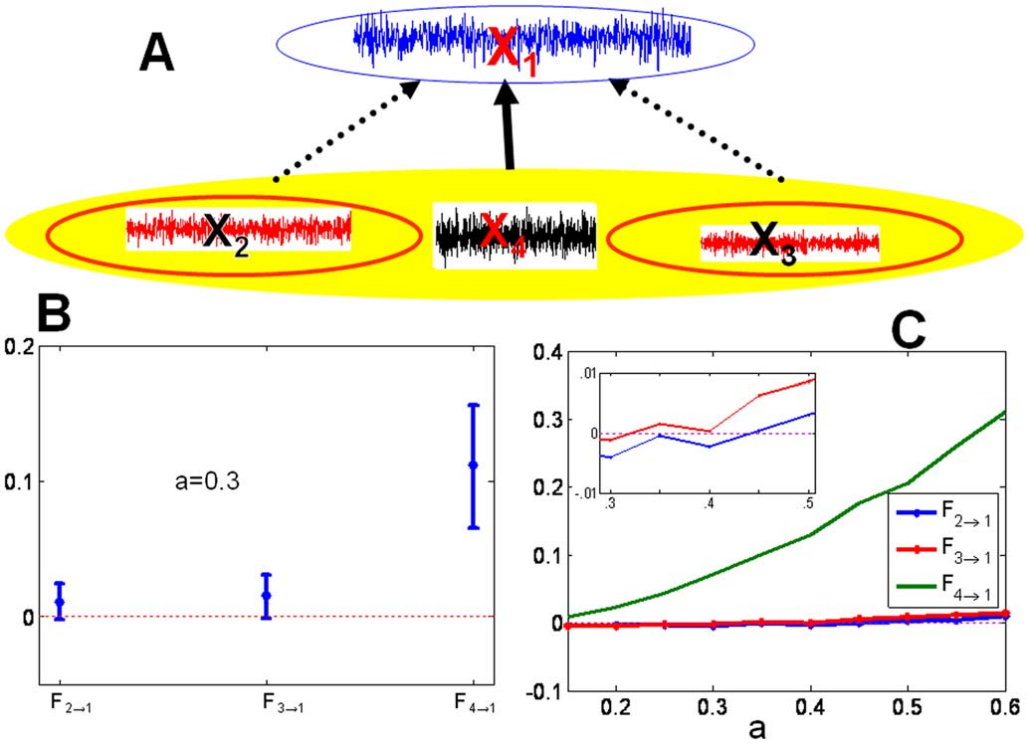
Weakly connected signals can have an overall significant effect. (A) The time courses of 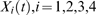 with 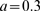. (B) The average value and its confidence interval of the Granger causality in example 2 when 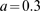. There are no causal relations between 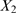 and 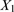, and 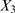 and 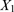, but the causal relationship between 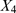 and 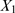 is significant. (C) The lowest value of the confidence intervals as a function of 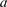. The inset shows the increasing, as one would expect, values of 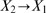 and 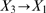 but on too small a scale to be significant.

Consider the following model
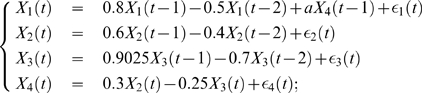
where 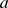 is a constant, 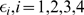 are independent standard normal random variables. The time courses of 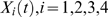 with 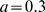 are shown in Fig. 3(**A**) . The parameter 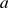 allows us to control how much influence a combination (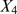) of 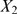 and 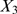 has on 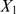.

In Fig. 3B, the mean values of the Granger causality together with their 3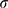 confidence intervals are depicted. Treating 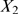 and 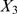 as a single units shows no interaction to 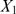. However their combination as 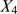 shows a significant interaction with 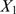.

In Fig. 3C, we plot the lowest value of the confidence interval of Granger causalities as a function of 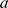. By construction, the contribution of 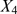 to 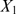 is 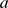 whereas the contributions of 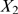 and 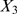 are 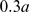 and 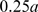 respectively. Thus, even with 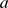 relatively big, 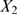 and 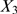 have little influence on 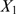, which is not the case for 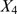. This is captured by the complex Granger causality: fig. 3C shows very small values for 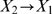 and 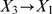 even for large values of 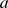 whereas 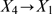 increases very rapidly.

### Simulated data: partial complex interaction

Indirect connections can produce spurious links between groups of interest. We have extended the method further with *partial complex Granger causality*, which estimates the complex Granger causality while reducing the influence of a third group.

#### Example 3

We modify example 1 to the following model
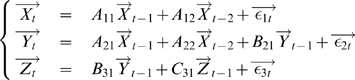
where 
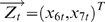
, 
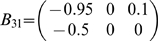
, 
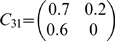
.

**Figure 4:**
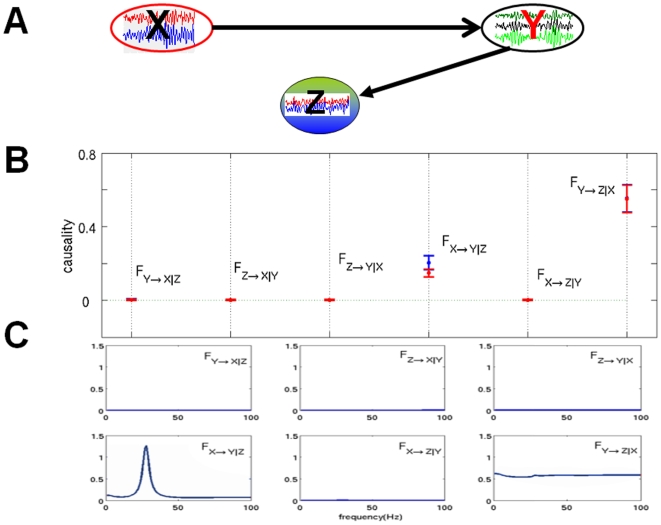
Partial Granger causality discards indirect connections. (A). Simulated time series and the underlying causal relationships considered in example 3. 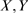 and 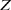 are multi-dimensional. (B) Blue and red error bars are defined as in figure 2. (C) Corresponding spectra in the frequency domain. The partial complex Granger causality and its 95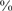 confidence intervals after 1000 replications.

We perform a simulation of this system to generate a data set of 2000 data points with a sample rate of 200 Hz. The time series are plotted in Fig. 4 (**A**). From the model, we see that only 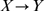 and 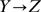 are direct interactions, as depicted in Fig. 4 (**A**). Fig. 4(**B**) presents a comparison between the time domain partial complex Granger causality and the frequency domain partial complex Granger causality. They are both in very good agreement. Fig. 4 (**C**) shows the results obtained in the frequency domain and reveals at which frequencies the signals interact.

Applying a complex Granger causality from 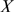 to 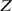 gives a value of 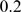, which is misleadingly high given the indirect nature of their connection. In contrast, considering the partial complex Granger causality 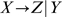 removes the influence of 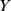 and gives a more accurate value of 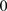: 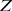 can be completely explained in terms of 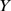 alone.

### Complexes in the yeast cell-cycle

We now apply our method to the binding interactions of proteins during the cell cycle of the budding yeast *Saccharomyces cerevisiae*. A gene can be activated by a combination of multiple transcription factors (a complex) and our aim is to show that grouping those transcription factors that act together strengthens the connection to their target genes. We use the microarray data produced for a study of the yeast's cell cycle ([13], [14]). We selected 12 time courses corresponding to either transcription factors or cell-cycle related genes. Among the 5 transcriptions factors, we know that some belong to the same complexes (MBP1 and SWI6, SWI4 and SWI6, from MIPS, [15]) and we expect their combination to have a stronger effect than when considered individually.

**Figure 5:**
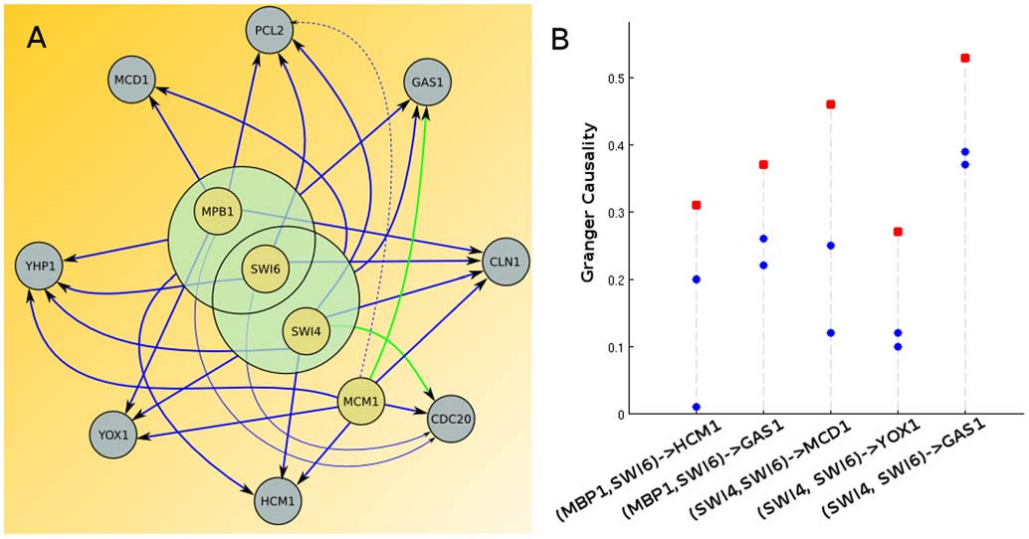
Complexes in a regulatory network. A) Inferred regulatory network of 12 genes known to participate in the yeast cell-cycle. Thick lines (blue if the interaction is documented, green if only potential according to YEASTRACT) are correct inferences. Thin lines are wrong inferences, with a dashed line representing a missed connection and a solid line representing a wrongly attributed connection. Yellow nodes denote target factors, green nodes complexes and blue nodes target genes. B) Improvement of the connection when complexes are considered. Blue dots represent the Granger causality from one member of the complex to the target gene, red squares represent the Granger causality from the complex to the target gene. Note that this hypergraph is not to be read as a power graph ([35]) as a connection from a complex to a target gene does not imply significant interactions from each of the subset elements to the target.

In order to test this claim, we apply Granger causality on all pairs (Transcription Factor, Gene) and (Complex, Gene). The inferred network is compared against the true network, built from the up-to-date data available on the curated YEASTRACT database ([16]). The resulting network is shown on figure 5. The program missed only one interaction (thin dashed line) and most of the calculated connections are true positives (thick lines) - they do exist in the true network, either from documented evidence (blue lines) or marked as potential (green lines) in YEASTRACT. The thin blue lines represent false positives, *i.e.* links suggested by the causality measure but not found in the literature. Most of the network is very close to the true network.

As seen on figure 5B, using a complex can greatly improve the strength of the interactions between transcription factors and target genes. Connections that could have been erroneously discarded at low threshold are more likely to be kept once the complex is considered.

### Directionality of connections between brain regions

In neuroscience, it is often of great importance to uncover connections between brain regions. Since most techniques are based on the interactions between individual signals, a workaround is usually to average them over a region of interest (see [17] for an example on fMRI data) beforehand. This can be misleading as a (weighted) average cannot capture the overall effect of individual interactions. It is especially true when spatial resolution is very high: interactions between groups of neurons are much more informative than those between individual neurons. In this section, we consider the neuronal activity of the left and right inferior temporal cortex (IT) in a sheep's brain, before and during a visual stimulus. We compare three approaches for the investigation of directionality between the two hemispheres. We first take a pairwise approach, by computing the Granger causality between each possible pairs of signals from both hemispheres. We then use the average signals from each region. Finally, we use the complex Granger causality to directly calculate the causality between the two regions as a whole.

**Figure 6:**
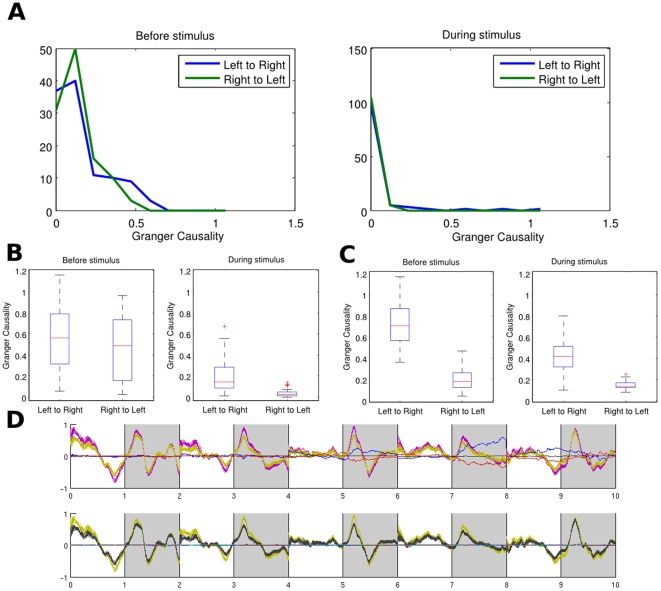
Group causality between brain regions A) Distribution of Granger causality between all 110 pairs of left and right signals. B) Distribution of Granger causality between region averages for each of the 40 experiments. C) Distribution of Complex Granger causality between the two regions for each of the 40 experiments. Each distribution is summarized by a boxplot showing its median (in red), as well as its first and third quartile (box). Smallest and largest values are shown with the outer bars and outliers are represented by red crosses. D) Signals from the left and right hemispheres for 5 experiments. Areas in gray denote the presence of the stimulus.

The recording was carried out when the sheep looked at a fixation point for one second and then an image (a pair of faces) for one second. The animal was handled in strict accordance with good animal practice as defined by the UK Home Office guidelines, and all animal work was approved by the appropriate committee. We have the recordings of 64 local field potentials in each region, sampled at a rate of 1000 Hz. Fig. 6D shows the signals from 5 experiments for both hemispheres. Fourty experiments were done with the same sheep, totalling 80’000 (40×(1000 ms +1000 ms)) time points for each signals. We selected the time series with significant variation (standard deviation 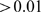). After this filtering, the left and right regions contain respectively 10 and 11 signals.

We first look at relations between individual signals. Figure 6A shows the distributions of the Granger causalities between all the 110 pairs of signals between the two regions. In both cases (before and during the stimulus), the curves are indistinguishable and the causality factors are low. No clear direction emerges from using single time-series.

Figure 6B shows the Granger causality between region averages. Before the stimulus, the connections from left ro right and right to left have very similar distribution, with such a large error over the 40 experiments that it makes the result inconclusive. During the stimulus, the connection from right to left vanishes, while the connection from left to right significantly decreases.

In constrast, using the complex Granger causality makes for a clearer picture, as seen in figure 6C. Here the causality is calculated between the two regions taken as multi-dimensional time-series. Before stimulus, there is an almost unidirectional flux of activity from left to right. This is still the case – if less pronounced – during the stimulus. This clear assymetry between the left and right hemispheres during face recognition has been reported in the litterature not only for sheep [18], [19] and ungulates but primates as well [20].

### A metabolic network

Metabolic networks consist of elaborate interdependent chemical reactions, whose rates are controled by enzymes. In this section, we show how Granger Causality can distinguish between the action of an enzyme and the action of a substrate. For clarity, we consider two canonical reactions rather than a whole network.

#### Example 4

Let us consider the following reaction:
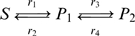



The corresponding dynamic system is as follows:
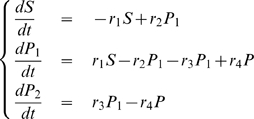
(33)


From this, we can show that 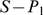 is a function of 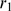 while 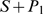 is not:

#### Example 5



**Figure 7:**
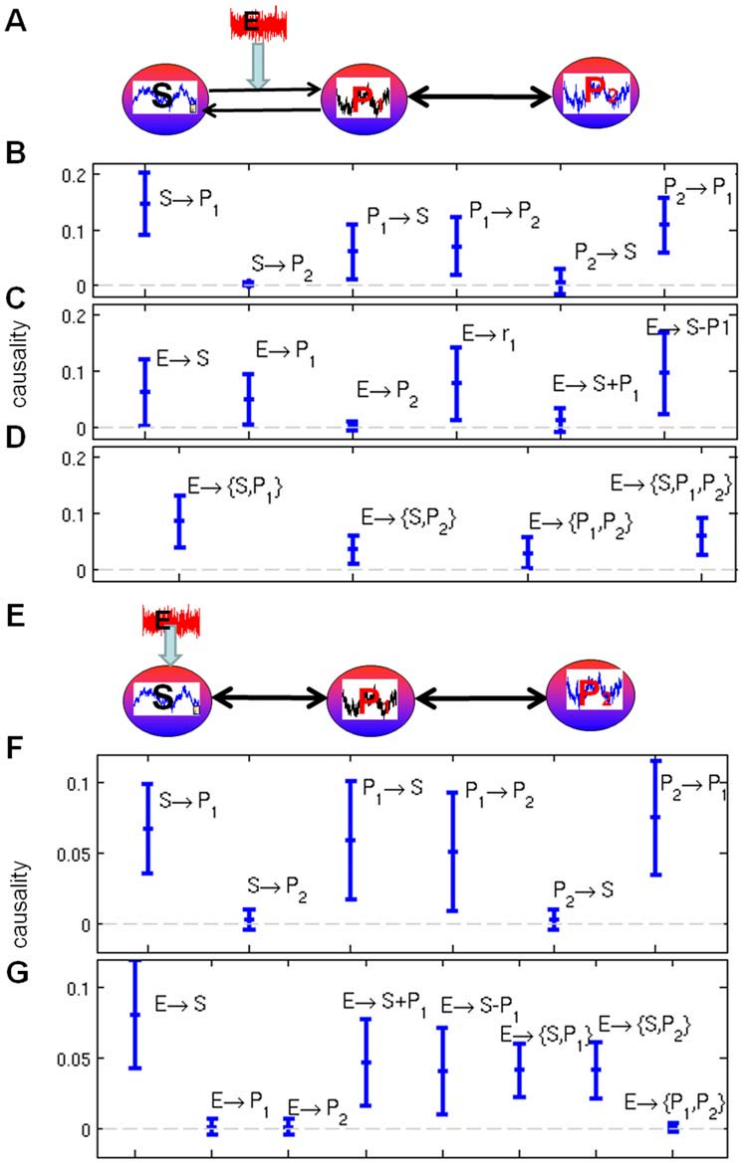
An enzymatic reaction (A): Time course of the three reactant 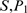 and 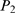 in Example 4. The enzyme 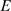 has direct influence on the reaction rate 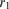. (B): Partial Granger causality between three reactants 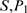 and 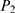 in Example 4. (C): Partial Granger causality from 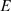 to other reactants in Example 4. (D): Partial Granger causality from 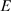 to complex of 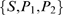 in Example 4. (E): Time course of the three reactant 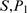 and 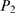 in Example 5. In this example, the enzyme 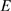 has direct influence on S. (F): Partial Granger causality between three substance 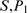 and 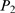 in example 5. (G): Partial Granger causality from 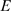 to other reactants and groups 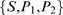 in example 5.

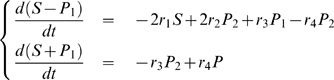
(34)Following the same reasoning we used in example 2, we should expect the Granger Causality from 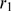 to 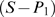 to be high and to 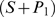 to be low. However, in pratice we don't have direct access to 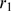. Now suppose that an enzyme 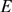 acts on the reaction rate 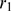 from 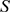 to 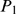 as 
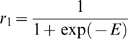
. We model the concentration of 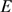 as 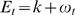 where 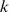 is a constant and 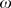 a normally distributed random variable. We generate the time courses of 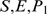 and 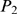 and compute the partial complex Granger causalities from 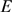 and from 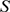. Fig. 7 shows the network and the calculated Granger causalities. The data were generated with 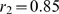, 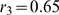, 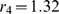, 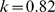 and 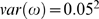. Using different parameters will produce similar results.

As in example 3, the partial causality is able to weed out indirect connections: for example, 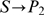, 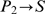 and 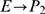 are all zero or very small. Conversely, direct connections are also recovered: 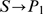, 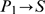, 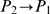 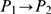 are high. But more interestingly, 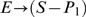 is also high – as high as the more obvious connection 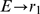 is in fact – and 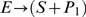 is low. In other words, 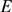 has the same causal characteristics than 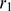 or 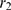 and we can conclude from the observed data alone that 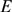 acts on the reaction rate between 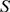 and 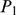, even though the reaction rate is not observed and the relation between 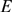 and 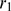 is non-linear.

Let us contrast this result with the case where 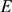 acts as a substrate and not an enzyme:
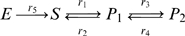



The corresponding dynamic system is:
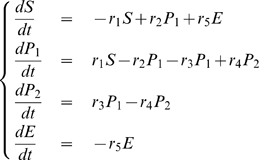
(35)Where 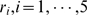 are constants. We set 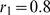, 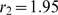, 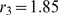, 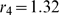, 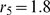 and generate the corresponding data. Fig.7F and G show the partial complex Granger causalities calculated from 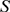,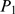, 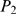 and 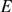. As in the previous example, indirect connections are correctly found to be zero: 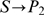, 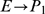, 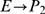 etc. Direct connections like 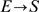, 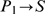, 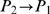 etc. have large values, which is expected. In this case however, we can reject the hypothesis that 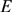 acts as an enzyme between 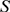 and 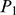 since 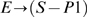 and 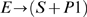 are equal and small.

In conclusion, it is possible to use partial complex Granger causality for uncovering the relations between elements of a metabolic network and avoiding false positives from indirect connections. But Granger causality can also detect interactions on reaction rates, that is, interactions on connections between elements, as has been demonstrated in this section.

### Impact of correlation on Granger causality

**Figure 8:**
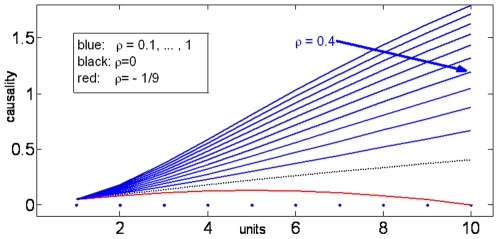
The role of correlation in the complex interaction. The Granger causality vs. units (N) for different cross-correlation coefficients 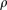 with a = 0.022.(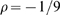 is the smallest possible value for the cross-correlations of 10 units.)

The complex Granger causality between a group and a target signal can be affected by the original signals' cross-correlations. Let us consider a model where 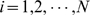 are identical random processes. The Granger causality from 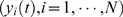 to their weighted sum 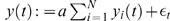 is 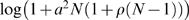 where 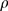 is the correlation coefficient between 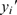
′s and 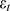 is normaly distributed. Fig. 8 illustrates how the complex interaction depends on the correlation. If the original signals are not correlated (black dashed line), taken as group they have increasingly higher interaction with 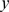 with the number of units. But this interaction is always higher the more positively cross-correlated they are. Conversely, negative cross-correlation reduces the interaction, all the way down to zero even though the target signal 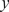 is made up of each of these signals by construction. Not surprisingly, collaborative activity enhances the interaction, but antagonistic activity reduces or even suppresses the interaction.

## Discussion

We have presented a study for the complex Granger causality. The time domain complex Granger causality and its frequency domain decomposition have been successfully applied to simulated examples and experimental data.

### An improvement over Partial Granger Causality

In [12], we have introduced the notion of partial Granger causality and successfully applied it to gene and neuron data. Although partial Granger causality is formally formulated for any dimension (see [12] Eq. 5), it leads to numerical instability when used on high dimensional data and we actually only restricted ourselves to the one-dimensional case. Partial Granger causality is defined as the ratio of the determinants of two theoretically positive definite matrices. In practice, however, these matrices often are only positive semidefinite due to the instability of the linear regression procedure. As a result, the determinants can reach very small values, even zero (since 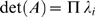) and easily produce very misleading results. Partial Complex Granger Causality uses the trace of matrices rather than their determinants and has proved much more stable for multi-dimensional data. Note that since trace and determinant are equal for one-dimensional signals, both definitions are equivalent in this case and results presented in [12] are obviously still valid.

Granger causality is always non-negative in the one-dimensional case. A natural question is whether this is is still the case with multi-dimensional signals. This would be equivalent to set an order in space of variance matrices. It turns out (see *e.g.* [21], p. 469) that it is possible to do so by setting that for two variance matrices 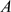 and 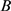, 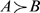 if and only if 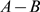 is positive semidefinite. However, we can easily see that if 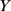 is a complex Granger cause of 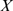, it does not imply that 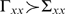.

### The importance of considering complex interactions

If we want to understand biological processes in details, at least two things are required: a large amount of accurate data and suitable computational tools to exploit them. Thanks to the continuing advances of bio-technology, we are now in a situation where a wealth of data is not only routinely acquired but also easily available (*e.g.* [22], [23] for microarray experiments, [24] for neurophysiology). Moreover, this trend is accelerating, with new technologies becoming available ([25], [26]). The challenge now is to develop the tools necessary to make use of this information.

One approach to uncover the relations between elements of a system is to use the statistical properties of the measurements to infer ‘functional’ ([27]) connectivity. This is the case for, for example, Bayesian networks ([28]), Dynamic Bayesian networks ([29]) or Granger causality networks ([30]. Typically, a global network is inferred from the connectivity from one element to another, or from one group of elements (‘parents’ in Bayesian Network settings) to a single one. This approach has produced informative results ([31], [32]) and is a very active domain of research.

But there is a real need now to go one step further, beyond these types of interactions, and to be able to deal with more complex interactions to reveal the influence of an element on the connection between two others for example, or to detect group-to-group interactions. Such complex interactions are ubiquitous in biological processes: enzymes act on the production rate of metabolites, information is passed on from one layer of neurons to the next, transcription factors form complexes which influence gene activity etc. And such interactions will be missed out with current methods.

In this paper, we demonstrated that the newly defined complex Granger Causality is able to capture these kinds of connections. For example, we showed that considering the effect of transcription factors improves network inferences in the case of the yeast-cell cycle data (Fig. 5). The method was also able to detect the effect of the enzyme in a metabolic reaction (Fig. 7) and to give a clearer and more principled picture of brain area interactions than simple averaging (Fig. 6). Having defined a measure to quantify these processes is a crucial step towards deducing the complete mechanism of a biological system. The next challenge, however, is to come: how to define the correct/relevant grouping.

### Future challenges for systems biology

Consider a network of 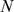 units (genes, proteins, neurons etc.). We intend to reveal all interactions in the network, this is the driving force behind the current systems biology approach ([33]). The belief is that the network interactions are the key for understanding many meaningful biology functions: from various diseases to brain function. For a network of 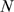 units, we might plausibly assume that there are 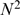 pairwise interactions (including self-interactions). Furthermore, a biological network is usually sparse and the total number of interactions should be much smaller. Hence, with simultaneously recorded data at 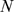 units, we hope to be able to recover all interactions. Here we point out, however, that the number of actual interactions should be of order 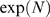, since all possible subsets (groups) of size 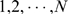 should be taken into account. This leads to an NP hard problem and a direct approach is bound to fail to reveal all interactions. The search space is now much bigger: we are not looking for the correct directed acyclic graph, or even graph but the correct hypergraph ([34]). Is a systems biology approach which would require to reveal all interactions including complex interactions reported here really feasible?
